# Axial Strength of Eccentrically Loaded FRP-Confined Short Concrete Columns

**DOI:** 10.3390/polym12061261

**Published:** 2020-05-31

**Authors:** Cheng Jiang, Yu-Fei Wu

**Affiliations:** 1 School of Engineering, RMIT University, 376-392 Swanston St, Melbourne, VIC 3001, Australia; 2 Department of Civil and Environmental Engineering, The Hong Kong Polytechnic University, Hung Hom, Kowloon 999077, Hong Kong, China; c.jiang@polyu.edu.hk; 3 Guangdong Provincial Key Laboratory of Durability for Marine Civil Engineering, Shenzhen University, Shenzhen 518060, China

**Keywords:** FRP, concrete, confinement, eccentric load, axial strength, column

## Abstract

This paper presents an experimental program that includes 78 fiber reinforced polymer (FRP)-confined square concrete columns subjected to eccentric loading. The degradation of the axial strength of FRP-confined short concrete columns due to the load eccentricity is investigated in this work. A larger load eccentricity leads to a greater decrease in the axial strength. From the test results, it is found that FRP confinement can cause less strength degradation compared with that of unconfined concrete specimens. For FRP-confined square concrete specimens, the strength enhancement due to FRP confinement increases with increasing load eccentricity. However, the increasing load eccentricity decreases the confinement efficiency for FRP-confined circular concrete specimens. The relationship between the strength of eccentrically loaded FRP-confined square columns and their corner radii is evaluated.

## 1. Introduction

Fiber reinforced polymer (FRP) jacketing or confinement has been widely adopted and used in rehabilitation work on concrete structures [1,2,3,4]. The confinement can not only enhance the strength and ductility of a concrete structure [5,6] but also increase the bond between the concrete and internal steel reinforcement [7,8,9]. In addition to the structural rehabilitation, the concept of FRP confinement has also been adopted in the constructions of new structures, such as the applications of concrete filled FRP tubes [10,11,12]. Various models for concrete columns strengthened with FRPs under concentric loading have been proposed in the past three decades. These stress-strain and strength models have covered different sections of circular (e.g., [13,14,15,16,17,18,19]), square (e.g., [20,21]), rectangular (e.g., [22,23,24]), and cross-sectional unified (e.g., [25,26,27]) types. In practical engineering applications, most existing concrete columns with noncircular cross-sections are subjected to eccentricity loading. However, concentrically loaded models have usually been used to analyze cases with eccentrically loading. It is well established in the literature that the load eccentricity affects the confinement behavior of FRP jacketed concrete columns [28,29,30,31] because the eccentric loading causes a variation in the confinement pressure across the section, thereby leading to a stress state that differs from that under concentric loading for a certain axial strain. 

Many experimental investigations on eccentrically loaded FRP confined columns have been carried out on circular columns [30,32,33,34,35,36,37,38,39,40,41] and noncircular columns [28,42,43,44,45,46,47,48,49,50]. Other investigations [29,31,51] focused on finite element modeling (FEM) to investigate the effect of the load eccentricity on FRP-confined concrete columns. It is found that the eccentric loading highly influences the mechanical behaviors of FRP-confined concrete. There is no doubt that the global response has a degradation trend due to load eccentricity. More recently, the effect of load eccentricity on the stress-strain relationship for cross-sectional analysis was deeply investigated and discussed. For example, Maaddaw [28] developed a simple theoretical stress-strain model considering load eccentricity, which has a reduction effect on the stress-strain relationship. However, other researchers found that load eccentricity enhanced the stress-strain behavior from both experimental [30] and numerical [31] analyses. 

Overall, the available tests on eccentrically loaded FRP-confined concrete columns are limited in the open literature, especially for square cross-sections. As the confinement mechanism for square columns is different with that of circular columns, it is necessary to clearly understand the relationship between column section and confinement efficiency under eccentric loading. This work aims to investigate the degradation of the axial load capacity due to the load eccentricity on FRP-confined square and circular short concrete.

## 2. Experimental Program 

The experimental program included 78 square confined columns with different FRP confinements (32 specimens without confinement, 32 specimens with 1-ply FRP confinement, and 14 specimens with 2-ply FRP confinement), corner radius ratios (12 specimens with 15 mm, 42 specimens with 30 mm, 12 specimens with 45 mm, and 12 specimens with 60 mm), and a full range of load eccentricities (12 specimens each for 0, 10, 20, 30, 40 and 50mm, and 6 specimens with 60 mm). The details of the specimen design are given in Table 1. All the specimens were tested in the Heavy Structure Testing Laboratory in the City University of Hong Kong. 

**Figure 1:**
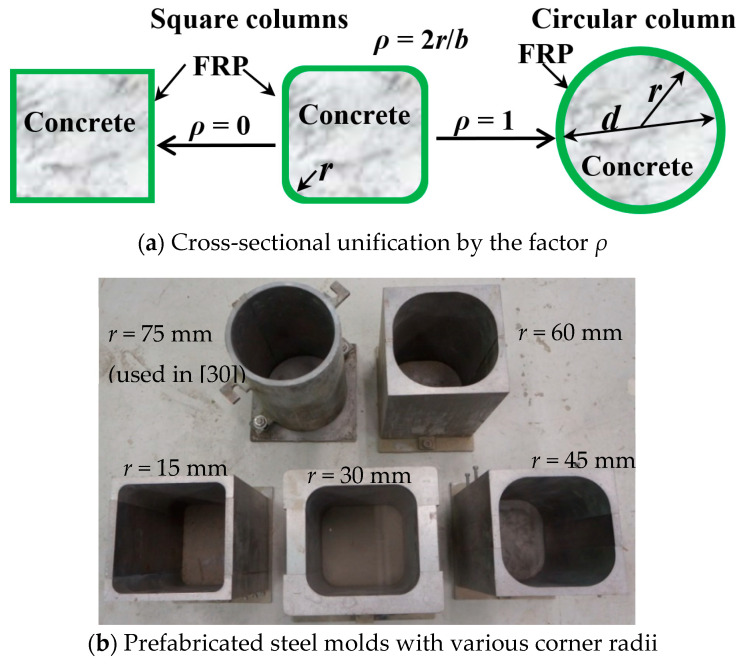
Cross-sections in this work: (**a**) Cross-sectional unification by the factor *ρ*; (**b**) Prefabricated steel molds with various corner radii.

All the specimens were designed with a width of 150 mm (*b*), a depth of 150 mm (*d*) and a height of 300 mm with different corner radii (*r*). To independently study the behaviors of the confined concrete, no internal reinforcement was used for the specimens. The corner radius ratio *ρ* is a key factor that affects the confining effect of FRP wrapping and is determined as [25,26,27,52]:

*ρ* = 2*r*/*b* (1)

where *b* is the length of the side for square cross-sections and the diameter for circular sections. The effect of the corner radius ratio *ρ* and the steel molds for concrete casting can be found in Figure 1.

As the specimens in this work could not be cast with one single batch of concrete, they were carefully grouped to minimize the unavoidable differences caused by the variations between the concrete properties of different batches. Specimens with the same FRP layers but different load eccentricities were grouped in one batch. Hence, the variation in the eccentricity could be isolated from the variation due to other factors.

A carbon fiber reinforced polymer (CFRP) was used for the FRP wrapping, which had a nominal thickness of 0.167 mm. A two-part Sikadur-300 was used as the saturant resin. Flat coupon tensile tests were conducted following ASTM D3039 [53] to determine the mechanical properties of the CFRP sheets. The ultimate strength, tensile modulus and ultimate strain of the CFRP sheets in this work were 4192 MPa, 254 GPa, and 1.84%, respectively. The CFRP sheets were wrapped around the specimens using the manual lay-up procedure in the hoop direction. Each CFRP jacket had a single overlap of 150 mm in length. 

For the specimen IDs in Table 1, the number after the letter R represents the corner radius with units of mm; subsequently, the two identical specimens are separated into two different groups identified by the letter A or B in the specimen IDs for the specimens with 30 mm of corner radius; the second figure of specimen ID (0, 1 or 2) denotes the number of CFRP layers; and the remaining indicators, E0, E10, E20, E30, E40 or E50, represent a load eccentricity of 0, 10,20, 30, 40 and 50 mm, respectively. For example, R45B2E30 indicates the FRP-confined square concrete specimen with a corner radius of 45 mm in group B, which was applied with 2-ply CFRP sheets and a load eccentricity of 30 mm.

**Figure 2:**
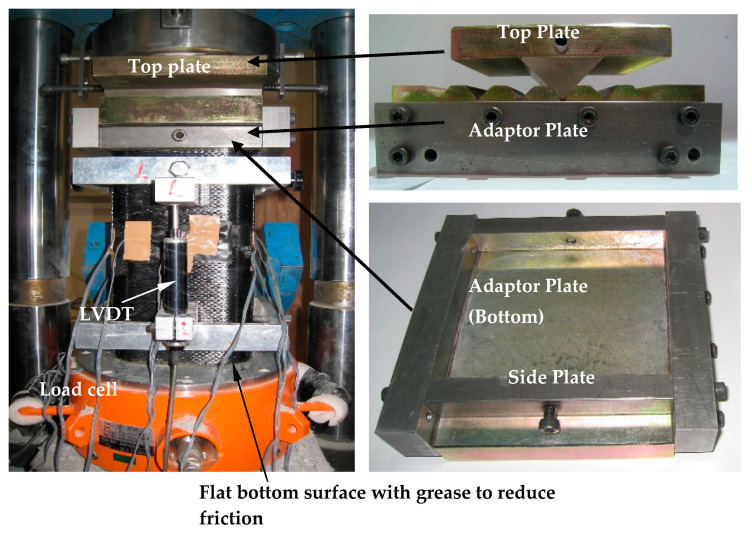
Test setup.

All the specimens were tested after 28 days of curing. The test setup is shown in Figure 2. Eccentric loading was applied by a knife edge at the top of the specimen (Figure 2). The specimen was placed on a flat and greased plate at the bottom so that friction at the end was minimized. The vertical displacements of the specimen at both the compression and tension sides were measured by two 50 mm-travel linear variable differential transformers (LVDTs), which were mounted on an aluminum frame with a 200 mm gauge length in the middle. A total of 75 mm was left between the specimen surfaces and the LVDTs. The specimens were tested under monotonically increasing loading until failure, controlled with a displacement rate of approximately 0.3 mm/min. The load was measured by a load cell placed at the bottom of the specimen (Figure 2). 

## 3. Test Results and Discussion

### 3.1. Failure Modes and Cross-Sectional Strength

**Figure 3:**
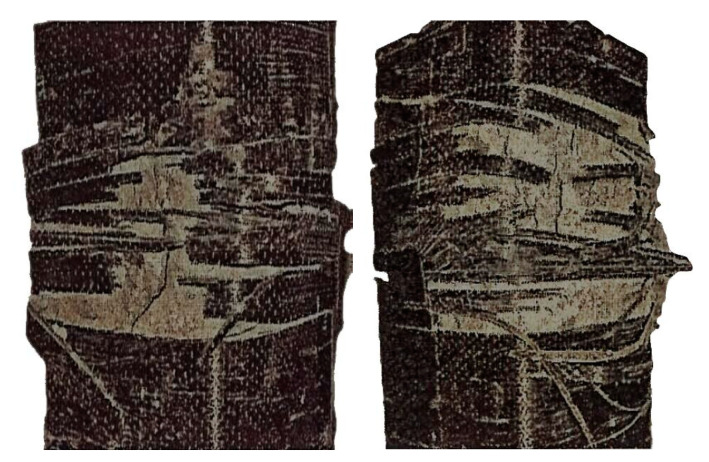
Typical failure mode.

For unconfined concrete specimens with concentric loading, the concrete mainly split longitudinally on the side surface, and the cone appearance could also be found on the failure surface. For unconfined columns with relatively larger load eccentricities, the failure of the specimens was generally marked by concrete crushing at the compression side at or near the mid-height of the specimen, and a horizontal crack at approximately mid-height or at the top of the column was clearly observed on the tensile side. For the columns with the largest eccentricity of 60 mm, the specimens crushed at the top of the specimen on the compression side. The typical images of failure modes of the tested FRP-confined square short columns are shown in Figure 3.

**Figure 4:**
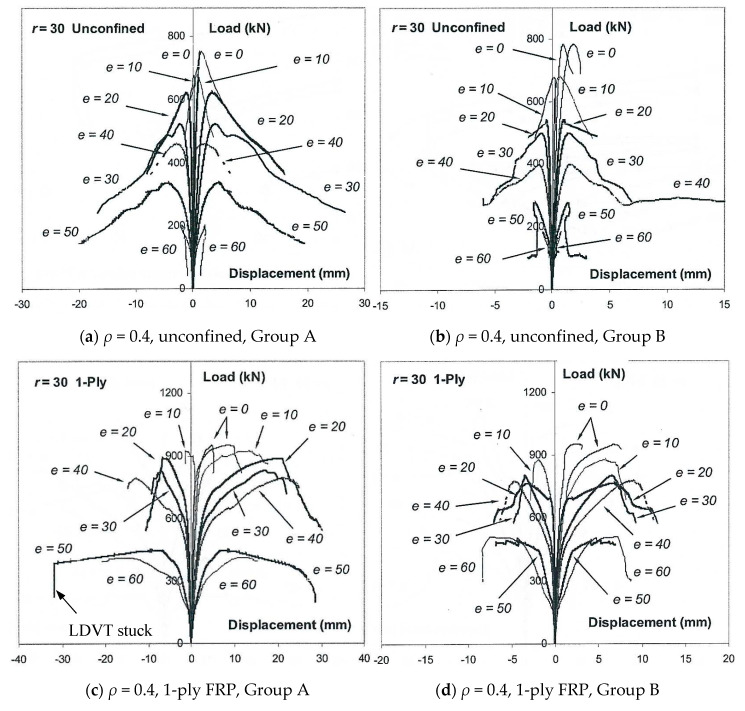

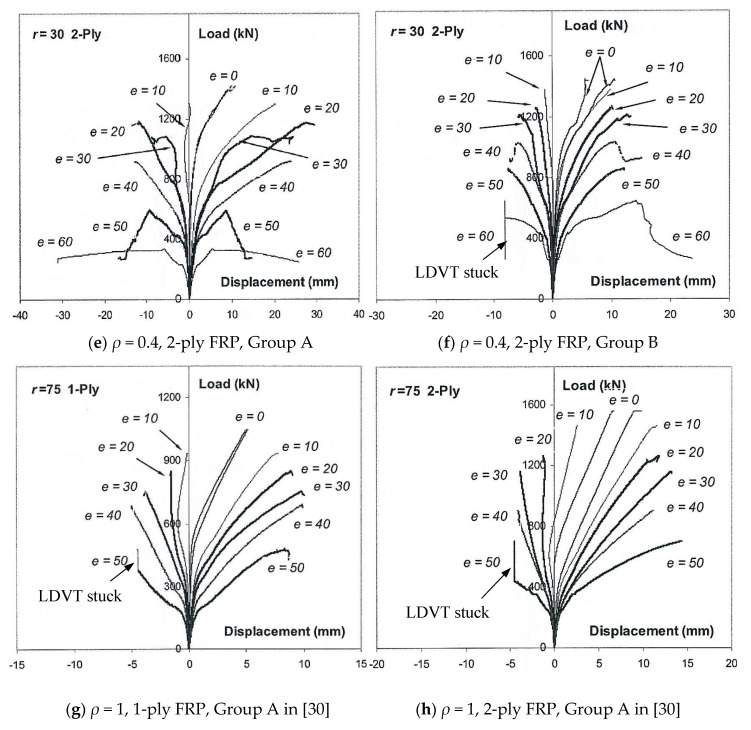
Typical load-deformation curves: (**a**) *ρ* = 0.4, unconfined, Group A; (**b**) *ρ* = 0.4, unconfined, Group B; (**c**) *ρ* = 0.4, 1-ply FRP, Group A; (**d**) *ρ* = 0.4, 1-ply FRP, Group B; (**e**) *ρ* = 0.4, 2-ply FRP, Group A; (**f**) *ρ* = 0.4, 2-ply FRP, Group B; (**g**) *ρ* = 1, 1-ply FRP, Group A in [30]; (**h**) *ρ* = 1, 2-ply FRP, Group A in [30].

The failure of all FRP-confined specimens was always caused by FRP rupture, and no delamination of the FRP at the overlapping zone was observed. Clicking sounds could be heard during the loading stage, and the failure occurred suddenly with an explosive sound. The fracture of the CFRP jacket was mainly near the corner of the compression side due to the stress concentration. On the tensile side, horizontal cracks along the fiber hoop direction were observed at mid-height or in adjacent areas. Furthermore, crushing of the concrete core occurred in the specimens with a relatively large load eccentricity. The test results for the axial strength, which refer to the peak load for a section in the tests (*N_e_*), of all the specimens are listed in Table 1. The typical load-displacement curves for eccentrically loaded FRP-confined square columns in this work and FRP-confined circular columns reported in [30] are illustrated in Figure 4. Each specimen in Figure 4 has two curves from two LVDTs on the compression side and tension side. Similarly to the circular columns, the degradation due to the load eccentricity is substantial, as observed in Figure 4. The sudden load loss or drop in the load-displacement curves indicates the occurrence of FRP rupture.

### 3.2. Effect of the Load Eccentricity and FRP Confinement

The strength factor *N_e_*/*N_c0_* is defined and analyzed to study the effect of the load eccentricity on the degradation of the axial strength, where *N_e_* is the load capacity of the sections for FRP-confined concrete specimens with/without load eccentricity and *N_c0_* is the load capacity for concentrically loaded unconfined concrete specimens. This type of load capacity comparison has been widely adopted in confined concrete analysis [54].

**Figure 5:**
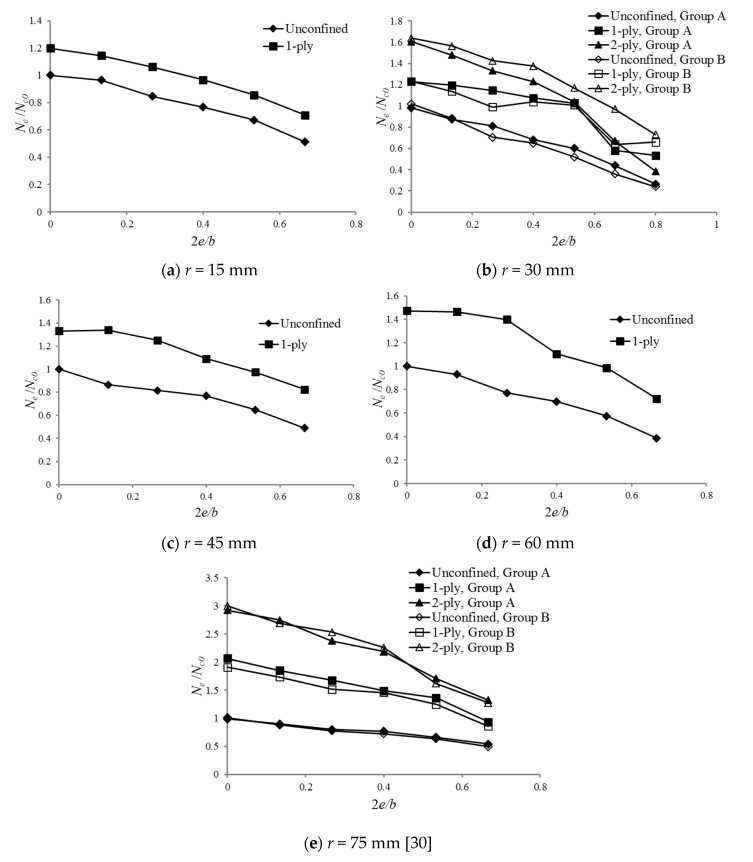
Effect of the load eccentricity on *N_e_*/*N_c0_*: (**a**) *r* = 15 mm; (**b**) *r* = 30 mm; (**c**) *r* = 45 mm; (**d**) *r* = 60 mm; (**e**) *r* = 75 mm [30]*_._*

A nondimensionalized term 2*e*/*b* is adopted to show the magnitude of the load eccentricity. By comparing the values of *N_e_*/*N_c0_*, it is obvious that the axial strength decreases with increasing load eccentricity, as shown in Figure 5. When the eccentricity is small (e.g., 10 mm or 2*e*/*b* = 0.13), the cross-sectional strength reduction degree is limited and is occasionally close to 0. However, when the load eccentricity increases, the decrease in the axial strength is more significant. Additionally, a greater confinement (or number of FRP layers) results in a higher strength.

**Figure 6:**
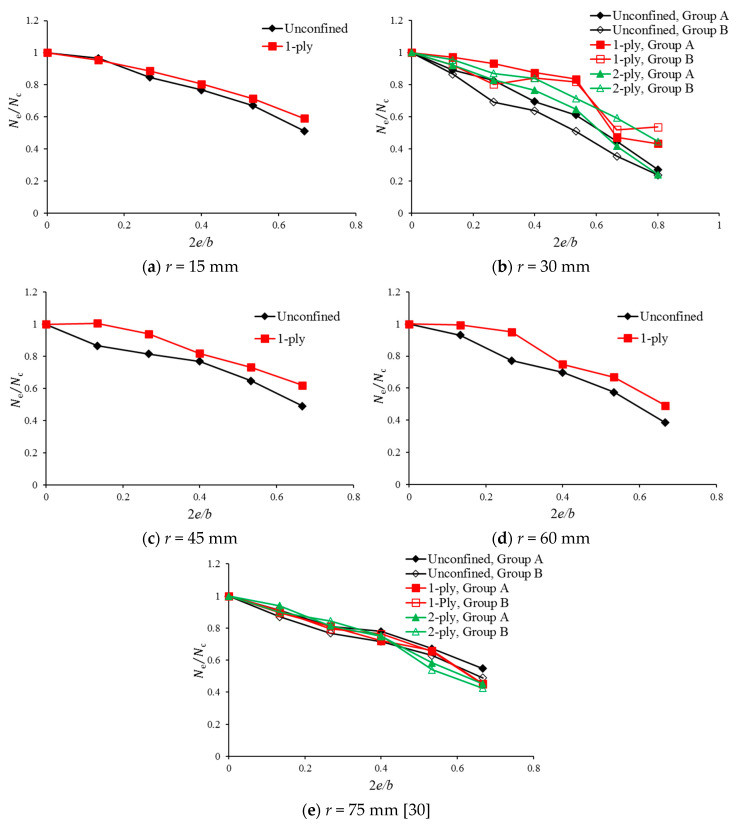
Effect of the load eccentricity on *N_e_*/*N_c_*: (**a**) *r* = 15 mm; (**b**) *r* = 30 mm; (**c**) *r* = 45 mm; (**d**) *r* = 60 mm; (**e**) *r* = 75 mm [30].

To better understand the strength degradation due to the load eccentricity, another comparison of *N_e_*/*N_c_* is carried out by modifying the results in Figure 5, where *N_c_* is the axial strength for concentrically loaded specimens with the same confinement level as that of *N_e_*. The results are shown in Figure 6. It can be concluded that the degradation of FRP-confined circular columns due to the load eccentricity (Figure 6e) is insensitive to the FRP confinement. However, for square columns (Figure 6a–d), FRP confinement can cause less axial strength degradation compared with that of unconfined concrete specimens. Different confinement levels (i.e., 1 layer and 2 layers) do not have a significant influence on the strength degradation, as can be observed in Figure 6b. 

**Figure 7:**
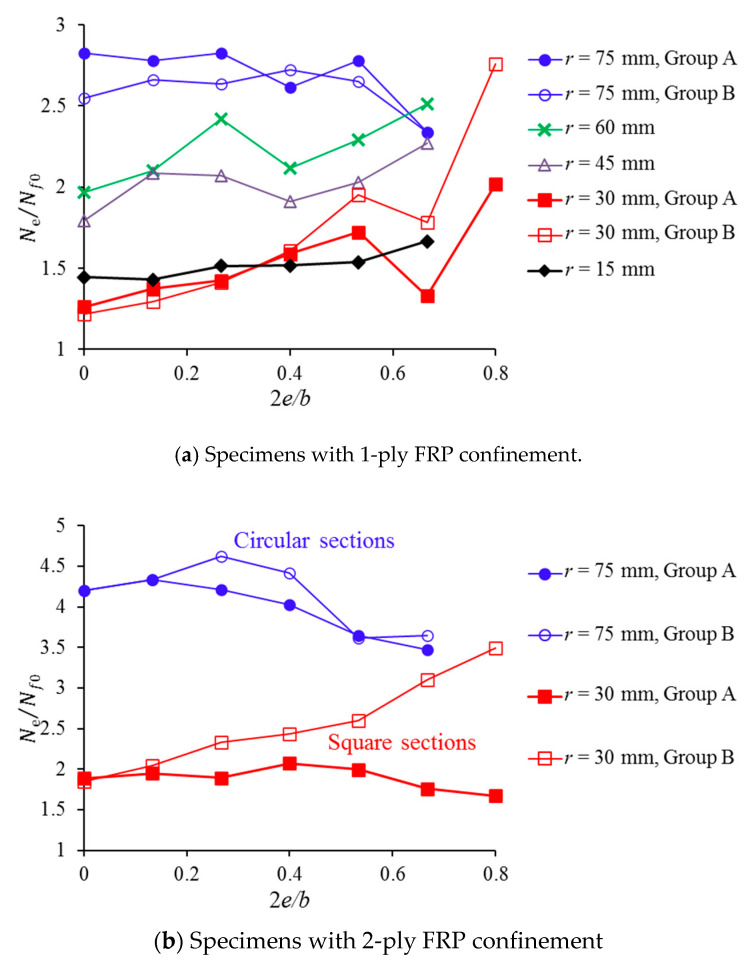
Effect of the load eccentricity on *N_e_*/*N_f_*_0_: (**a**) Specimens with 1-ply FRP confinement; (**b**) Specimens with 2-ply FRP confinement_._

The contribution from the FRP confinement can be further investigated by studying the effect of the load eccentricity on *N_e_*/*N_f_*_0_, where *N_f_*_0_ is the axial strength for unconfined concrete with the same load eccentricity as that of *N_e_*. The comparison results are shown in Figure 7. The confinement efficiency can be easily revealed for the term *N_e_*/*N_f_*_0_. A high value of *N_e_*/*N_f_*_0_ represents a higher efficiency due to the FRP confinement because this ratio represents the strength enhancement by the FRP confinement. For most FRP-confined square concrete specimens, it can be found in Figure 7 that the value of *N_e_*/*N_f_*_0_ increases when the load eccentricity increases. However, the increasing load eccentricity decreases the confinement efficiency for FRP-confined circular concrete specimens. This is reasonable because the circular section has less compressive area than that of square columns for a fixed load eccentricity or natural axis. This trend is more significant for a large eccentricity because of the small height of the natural axis.

### 3.3. Effect of the Corner Radius

When 2*e*/*b* = 0, which means the concrete is under concentric loading, a greater corner radius can result in a higher value of *N_e_*/*N_f_*_0_, as shown in Figure 7. An exception to this trend is the case where *r* = 15 mm (Figure 7a). This is because the specimens with *r* = 15 mm have the lowest unconfined concrete strength, as shown in Table 1. The lower concrete strength leads to a higher confinement effect for the fixed FRP wrapping layers.

The reduction in the confinement effectiveness due to the corner radius of an FRP-confined square-shaped column under concentric loading can be accounted for with an accurate shape factor *k_s_* [24,25,26] as:

*k_s_* = (2*r*/*b*)^0.72^ (2)

However, Equation (2) was proposed for the ultimate strength of the stress-strain relationship of FRP-confined concrete. Jiang et al. [55] extended and summarized a general relationship for the influence of the corner radius on the structural indexes (e.g., axial strength and plastic hinge length) of FRP-confined concrete structures, as shown in Equation (3).

*S_sf_*/*S_s0_* = 1 + (2*r*/*b*)^0.72^(*S_cf_*/*S_c0_* − 1) (3)

where *S_sf_* and *S_s0_* are the structural indexes of FRP-confined square concrete and unconfined square concrete, respectively; *S_cf_* and *S_c0_* are the performances of FRP-confined circular concrete and unconfined circular concrete, respectively. If Equation (3) is applied to determine the axial strength of FRP confined concrete under eccentric loading, Equation (3) can be written as:

*N_e_*/*N_f0_* = 1 + (2*r*/*b*)^0.72^(*N_c-f_*/*N_c-0_* − 1) (4)

where *N_c-f_* and *N_c-0_* are the *N_e_* and *N_f0_* values for 2*r*/*b* = 1 (i.e., circular section), respectively.

**Figure 8:**
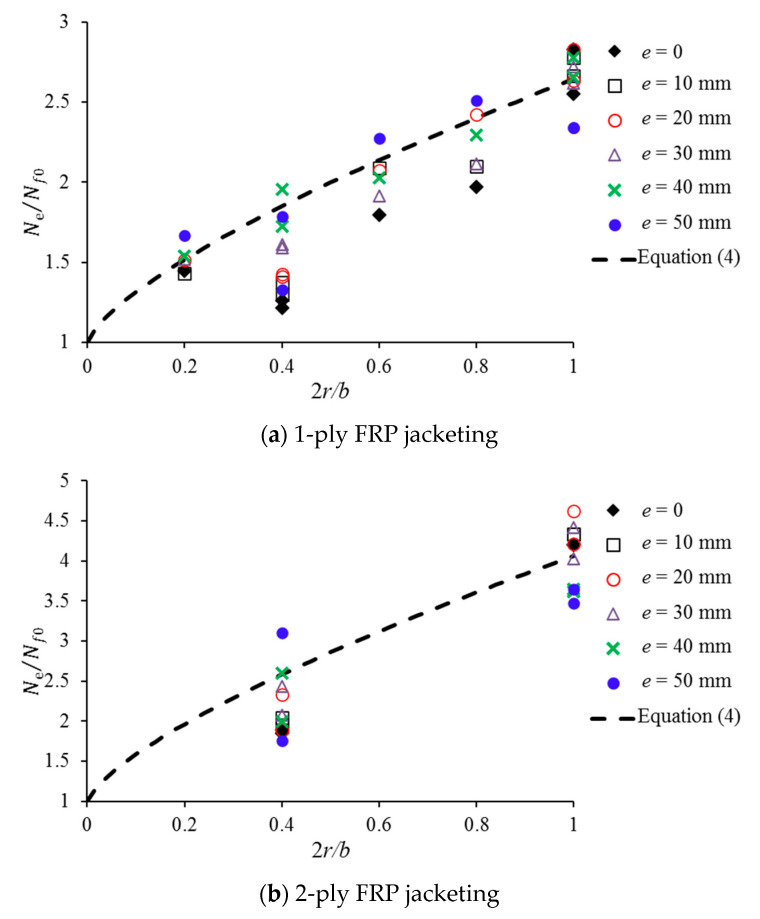
Effect of the corner radius on *N_e_*/*N_f_*_0_: (**a**) 1-ply FRP jacketing; (**b**) 2-ply FRP jacketing.

The *N_e_*/*N_f_*_0_ values for the various corner radii with a fixed number of FRP layers are plotted in Figure 8. It can be found from Figure 8 that the relationship for Equation (4) can basically describe the effect of the corner radius for FRP-confined concrete subjected to eccentric loading. Here, *N_c-f_*/*N_c-0_* in Equation (4) takes the average test values in Figure 8 in the analysis. This finding provides a foundation for developing an eccentricity-based stress-strain model of FRP-confined square columns, which requires future study. 

## 4. Conclusions

The compression tests on a total of 78 FRP-confined square concrete columns subjected to eccentric loading were reported in this work. The degradation of the axial load capacity due to the load eccentricity was investigated. After analyzing the test results, the following conclusion can be drawn.

(1) When the load eccentricity is small (e.g., 10 mm or 2*e*/*b* = 0.13), the axial strength reduction degree is limited and is occasionally close to 0. However, when the load eccentricity increases, the decrease in the axial strength is more significant.
(2) FRP confinement can reduce the axial strength degradation compared with that of unconfined concrete specimens. While different confinement levels (i.e., 1 layer and 2 layers) do not have a significant influence on the strength degradation.
(3) For FRP-confined square concrete specimens, the strength enhancement due to FRP confinement increases with increasing load eccentricity. However, the increasing load eccentricity decreases the confinement efficiency for FRP-confined circular concrete specimens. This is because the circular section has a much less compressive area than that of square columns for a fixed natural axis.
(4) The relationship between the structural behavior of FRP-confined square columns and their corner radii (Equation (4)) can basically describe the effect of the corner radius on the axial strength of FRP-confined concrete subjected to eccentric loading.

## Figures and Tables

**Table 1:** Details of the test specimens.

Specimen ID	*r* (mm)	*f_co_* (Mpa)	*e* (mm)	FRP Layers	*N_e_* (kN)	Specimen ID	*r* (mm)	*f_co_* (Mpa)	*e* (mm)	FRP Layers	*N_e_* (kN)
R15A0E0	15	25.6	0	0	571.8	R30B0E60	30	35.4	60	0	184.9
R15A0E10	15	25.6	10	0	551.8	R30B1E0	30	35.6	0	1	952.7
R15A0E20	15	25.6	20	0	483.8	R30B1E10	30	35.6	10	1	878.7
R15A0E30	15	25.6	30	0	438.8	R30B1E20	30	35.6	20	1	764.7
R15A0E40	15	25.6	40	0	383.8	R30B1E30	30	35.6	30	1	802.7
R15A0E50	15	25.6	50	0	292.9	R30B1E40	30	35.6	40	1	778.7
R15A1E0	15	30.9	0	1	826.7	R30B1E50	30	35.6	50	1	493.8
R15A1E10	15	30.9	10	1	788.7	R30B1E60	30	35.6	60	1	509.8
R15A1E20	15	30.9	20	1	732.7	R30B2E0	30	40.7	0	2	1448.5
R15A1E30	15	30.9	30	1	665.8	R30B2E10	30	40.7	10	2	1383.5
R15A1E40	15	30.9	40	1	589.8	R30B2E20	30	40.7	20	2	1261.6
R15A1E50	15	30.9	50	1	487.8	R30B2E30	30	40.7	30	2	1215.6
R30A0E0	30	35.4	0	0	753.7	R30B2E40	30	40.7	40	2	1034.6
R30A0E10	30	35.4	10	0	671.7	R30B2E50	30	40.7	50	2	858.7
R30A0E20	30	35.4	20	0	622.8	R30B2E60	30	40.7	60	2	645.8
R30A0E30	30	35.4	30	0	523.8	R45A0E0	45	26.8	0	0	555.8
R30A0E40	30	35.4	40	0	460.8	R45A0E10	45	26.8	10	0	480.8
R30A0E50	30	35.4	50	0	336.8	R45A0E20	45	26.8	20	0	452.8
R30A0E60	30	35.4	60	0	203.9	R45A0E30	45	26.8	30	0	426.8
R30A1E0	30	35.6	0	1	950.7	R45A0E40	45	26.8	40	0	359.8
R30A1E10	30	35.6	10	1	924.7	R45A0E50	45	26.8	50	0	271.9
R30A1E20	30	35.6	20	1	886.7	R45A1E0	45	36.1	0	1	997.7
R30A1E30	30	35.6	30	1	831.7	R45A1E10	45	36.1	10	1	1003.6
R30A1E40	30	35.6	40	1	793.7	R45A1E20	45	36.1	20	1	937.7
R30A1E50	30	35.6	50	1	447.8	R45A1E30	45	36.1	30	1	816.7
R30A1E60	30	35.6	60	1	411.8	R45A1E40	45	36.1	40	1	729.7
R30A2E0	30	40.7	0	2	1420.5	R45A1E50	45	36.1	50	1	617.8
R30A2E10	30	40.7	10	2	1308.6	R60A0E0	60	27.6	0	0	534.8
R30A2E20	30	40.7	20	2	1177.6	R60A0E10	60	27.6	10	0	497.8
R30A2E30	30	40.7	30	2	1086.6	R60A0E20	60	27.6	20	0	412.8
R30A2E40	30	40.7	40	2	918.7	R60A0E30	60	27.6	30	0	372.8
R30A2E50	30	40.7	50	2	591.8	R60A0E40	60	27.6	40	0	306.9
R30A2E60	30	40.7	60	2	340.8	R60A0E50	60	27.6	50	0	205.9
R30B0E0	30	35.4	0	0	782.7	R60A1E0	60	36.8	0	1	1052.6
R30B0E10	30	35.4	10	0	677.7	R60A1E10	60	36.8	10	1	1045.6
R30B0E20	30	35.4	20	0	541.8	R60A1E20	60	36.8	20	1	999.7
R30B0E30	30	35.4	30	0	498.8	R60A1E30	60	36.8	30	1	788.7
R30B0E40	30	35.4	40	0	398.8	R60A1E40	60	36.8	40	1	703.7
R30B0E50	30	35.4	50	0	276.9	R60A1E50	60	36.8	50	1	516.8

Note: *r* is the corner radius, *f_co_* is the unconfined concrete strength, *e* is the load eccentricity, and *N*_e_ is the axial load capacity (axial strength).

## Nomenclature

*b*	length of the side for square cross-sections and the diameter for circular sections
*d*	depth of the cross-section, = b for square cross-sections
*e*	load eccentricity
*f_co_*	unconfined concrete strength
*h*	height of the specimens
*k_s_*	shape factor for the corner radius, = (2*r*/*b*)^0.72^
*N_c_*	axial strength for the concentrically loaded specimens
*N_c0_*	axial strength for the concentrically loaded unconfined concrete specimen.
*N_c-0_*	*N_f0_* value for 2*r*/*b* = 1 (i.e., circular section)
*N_c-f_*	*N_e_* value for 2*r*/*b* = 1 (i.e., circular section)
*N_e_*	axial load capacity (or axial strength) of short columns
*N_f0_*	axial strength for the unconfined concrete specimens
*r*	corner radius
*S_c0_*	structural index of the unconfined circular concrete
*S_cf_*	structural index of the FRP-confined circular concrete
*S_s0_*	structural index of the unconfined square concrete
*S_sf_*	structural index of the FRP-confined square concrete
*ρ*	corner radius ratio, = 2*r*/*b*
